# Effect of processing methods on the nutritional content of three traditional vegetables leaves: Amaranth, black nightshade and jute mallow

**DOI:** 10.1002/fsn3.504

**Published:** 2017-08-19

**Authors:** Korotimi Traoré, Charles Parkouda, Aly Savadogo, Fatoumata Ba/Hama, Regine Kamga, Yves Traoré

**Affiliations:** ^1^ Institut de Recherche en Sciences Appliquées et Technologies Département Technologie Alimentaire 03 BP 7047 Ouagadougou Burkina Faso; ^2^ Laboratoire de Biochimie et d'Immunologie Appliquée Department of Biochemistry‐Microbiology Université Ouaga 1 Pr Joseph KI‐ZERBO Ougadougou Burkina Faso; ^3^ Asian Vegetable Research and Development Center Liaison office Cameroon Messa Yaoundé Cameroon

**Keywords:** *Amaranthus cruentus*, *Corchorus olitorius*, nutrient, processes, *Solanum scabrum*

## Abstract

The study assessed changes in nutritional content of some commonly consumed traditional vegetables subjected to postharvest processes. Amaranth (*Amaranthus cruentus* L.), black nightshade (*Solanum scabrum* Mill.) and jute mallow (*Corchorus olitorius* L.) leaves used as vegetables were subjected to blanching, boiling and drying. The proximate composition and β‐carotene content of fresh and processed leaves were determined. Amaranth, black nightshade and jute mallow leaves had 25.21%, 39.74% and 29.18% of protein, respectively. The β‐carotene levels were 16.40, 25.25 and 27.74 mg/100 g for black nightshade amaranth and jute mallow leaves, respectively. The ash content was 10.57% for black nightshade, 12.40% for jute mallow and 16.33% for amaranth. Processing methods caused decreases of β‐carotene and crude lipid content. Boiling for 30 min or more resulted in large loss of β‐carotene. Drying under shade resulted in less loss of β‐carotene than drying in cabinet at 50 and 60°C.

## 1 INTRODUCTION

Traditional African vegetables have values and properties that make them useful for farmers and consumers. Leafy vegetables are important sources of minerals, vitamins, fiber, amino acids and health‐promoting phytochemicals with antioxidant, antibiotic and anticancer and other nutraceutical properties (Grubben, 1976; Ihekoronye & Ngoddy, 1985; Oyenuga & Fetuga, 1975; Yang & Keding, 2009). Several of these vegetables are subjected to postharvest treatments of drying, blanching, or cooking to improve organoleptic properties and remove potential toxic components and for preservation purposes. Some processing techniques alter nutrient content of plants (Causeret, 1986; Frances, Thomas, & Gabriel, 2013; Shashi & Salil, 1996).

Traditional vegetables have been neglected by consumers because of introduction of exotic vegetables into markets (Remi, Ludovic, Pierre, & Hubert, 2005). In sub‐Saharan Africa, there is a diversity of leafy vegetables that are consumable (Remi et al., 2005). Despite this diversity, sub‐Saharan Africa has the lowest level of vegetable consumption in the world (AVRDC, 2003), and the highest level (15%) of malnutrition (FAO, 2015). Among these vegetables, amaranth (*Amaranthus cruentus* L.), black nightshade (*Solanum scabrum* Mill.) and jute mallow (*Corchorus olitorius* L.) are thought to benefit the human diet (Grubben et al., 2014; Mulokozi & Svanberg, 2003; Waliou, 2011). Promotion of traditional vegetables consumption can help reduce food insecurity and improve nutrition (Shashi and Salil, 1996; Kouame, Batchep, & Kamga, 2013; Grubben et al., 2014). Before consumption amaranth, black nightshade and jute mallow undergo postharvest processing that can affect their nutrient contents (Oboh, 2005). It is necessary to ensure that the processes applied do not affect the quality of the vegetables. Urbanization in developing countries and globalization have created a need for larger scale production of processed traditional vegetables with consistent quality since householders allocated less time to growing or preparation of vegetables for consumption (Kamga et al., 2013). Postharvest processes applied by producers is uncontrolled and leads to variation in product stability and quality. Despite the importance of traditional vegetables in the diet, understanding of postharvest processing of traditional vegetables is limited. The objectives of this study were to determine the nutritional content of traditional African vegetables and to assess effects of processing method on chemical composition of amaranth, black nightshade and jute mallow leaves.

## 2 MATERIAL AND METHODS

Ready to use fresh leaf samples were collected with producers from three production sites in Ouagadougou (Burkina Faso), and immediately transported to the laboratory for preparation. Samples were washed three times with tap water and drained. The leaves were subjected to the processing techniques of blanching, or boiling for 30 or 60 min, or shade drying and drying at 50 or 60°C using a gas convection dryer.

For blanching, 30 g of sample were immersed in hot water, 90–92°C for 2 min as described by James and Kuipers (2003). After treatment leaves were rinsed with cool tap water, drained, packaged in plastic bottles and kept frozen at −20°C, until analyses.

For boiling, 30 g of sample were subjected to boiling in 2 L of tap water and sub‐samples obtained at 30 and 60 min during boiling. The samples were cooled, packaged into plastic bottles and kept frozen at −20°C until analyses.

For drying, samples were dried in the shade and in a gas convection dryer at 50 and 60°C. During shade drying, temperature varied from 25.8 to 31.4°C, with a relative humidity value varied from 63% to 97%. Dried samples were packaged in opaque bottles and stored at laboratory temperature until analysis.

Content of moisture, ash, lipid, protein, carbohydrate and crude fiber in samples were performed according to international standards (NF V03‐707, 2000; ISO 2171, 2007; ISO‐659, 1998; NF V03‐050 NF, 1970; Montreuil & Spik, 1969; Deymie et al., 1981). β‐carotene content was determined by High Performance Liquid Chromatography as described by Craft (1992). Analyses were performed in triplicate for each sample.

The data were subjected to one‐way analysis of variance (ANOVA). Means were separated by the Tukey test based on the honest significant difference (HSD) test using the XLSAT software (ver. 7.5.2, Addinsoft. Paris, France).

## 3 RESULTS

The nutritional content of amaranth, black nightshade and jute mallow leaves differed (Table 1). Moisture content was similar for amaranth, black nightshade and jute mallow. There was less protein in leaves of amaranth and jute mallow and the highest value was in leaves of black nightshade. Crude lipid and total carbohydrates contents where similar for the crops. Fiber content was lowest for black nightshade and highest for jute mallow. The β‐carotene content of the black nightshade leaves was lower than for amaranth and jute mallow.

**Table 1:** Nutritional content of raw amaranth, black nightshade and jute mallow leaves (dry matter basis)

Crop	Moisture (%)	Ash (%)	Lipid (%)	Protein (%)	Carbohydrate (%)	Fiber (%)	β‐carotene (mg/100 g)
Amaranth	84.33 ± 1.26^a^	16.33 ± 1.27^a^	7.56 ± 0.37^a^	25.21 ± 2.63^b^	25.68 ± 2.36^a^	16.35 ± 2.29^ab^	24.25 ± 2.29^b^
Black nightshade	87.71 ± 1.61^a^	10.57 ± 1.12^b^	7.11 ± 1.22^a^	39.74 ± 1.92^a^	25.94 ± 5.05^a^	14.07 ± 0.39^b^	16.40 ± 0.08^c^
Jute mallow	84.49 ± 1.76^a^	12.40 ± 1.44^b^	6.64 ± 1.46^a^	29.18 ± 0.47^b^	27.88 ± 3.76^a^	20.86 ± 3.30^a^	27.71 ± 1.75^a^
*p*‐value	.105	.006	.601	.000	.718	.04	.000
*t* test	ns	**	ns	**	ns	*	**

Values in columns followed by the same letter are not significantly different.

ns, *, ** nonsignificant or significant at *p* < .05 or *p* < .01.

Blanching affected nutritional content of amaranth, black nightshade and jute mallow (Table 2). Moisture content of leaves increased due to blanching. There was no difference in the moisture content of the amaranth and black nightshade and jute mallow leaves, compared to the raw leaves. The ash content decreased by 2.61%, 1.73% and 3.83% for amaranth, black nightshade and jute mallow, respectively. There was no difference in protein content in amaranth and black nightshade leaves due to blanching. There was a difference between raw and blanched jute mallow leaves. Total carbohydrate content increased after blanching for amaranth and black nightshade. There was a decrease for jute mallow leaves. Blanching did not affect crude lipid content. There was a decrease in β‐carotene content due to blanching.

**Table 2:** Impact of blanching process on the nutritional content of the vegetables (dry matter basis)

Crop ×	Condition	Moisture (%)	Ash(%)	Protein (%)	Carbohydrate (%)	Lipid (%)	β‐carotene (mg/100 g)
Amaranth	Raw leaves	84.33 ± 1.26^a^	16.33 ± 1.27^a^	25.21 ± 2.63^b^	25.68 ± 2.36^a^	7.56 ± 0.37^a^	24.25 ± 2.29^b^
	Blanched	88.51 ± 1.39^a^	13.72 ± 0.72^a^	26.86 ± 2.75a	42.35 ± 9.83^a^	5.75 ± 0.16^a^	14.85 ± 0.09^b^
	*p*‐value	.070	.144	.496	.046	.086	.003
	*t* test	ns	ns	ns	*	ns	*
Black night shade	Raw leaves	87.71 ± 1.61^a^	10.57 ± 1.12^b^	39.83 ± 2.04^a^	29.04 ± 5.05^b^	7.60 ± 0.83^a^	16.39 ± 0.83^a^
	Blanched	91.50 ± 1.57^a^	8.84 ± 1.63^a^	40.60 ± 3.58^a^	60.69 ± 13.44^a^	7.28 ± 1.38^a^	11.86 ± 0.05^b^
	*p*‐value	.023	.204	.764	.013	.750	.000
	*t* test	*	ns	ns	**	ns	**
Jute mallow	Raw leaves	83.73 ± 1.64^b^	12.40 ± 1.44^a^	28.98 ± 045^a^	26.63 ± 4.36^a^	6.64 ± 1.46^a^	27.71 ± 1.75^a^
	Blanched	91.68 ± 0.11^a^	8.57 ± 1.56^b^	27.09 ± 0.49^b^	17.55 ± 2.25^b^	6.63 ± 0.17^a^	0.57 ± 0.09^b^
	*p*‐value	.003	.036	.003	.017	.952	.0001
	*t* test	**	*	**	*	ns	**

Values in columns followed by the same letter are not significantly different.

ns, *, ** nonsignificant or significant at *p* < .05 or *p* < .01.

Boiling affected nutritional content of amaranth, black nightshade, and jute mallow leaves (Table 3). Moisture content increased from the onset until 30 min for all crops and decreased at 60 min. The values were higher than the moisture content in raw leaves for all crops. There was no difference between ash content in boiled and raw leaves. The ash content in leaves boiled for 30 min was lower than leaves boiled for 60 min.

**Table 3:** Effect of boiling on nutritional content of the vegetables (dry matter basis)

Crop	Condition	Moisture (%)	Ash (%)	Proteins (%)	Carbohydrates (%)	β‐carotene (mg/100 g)
Amaranth	Raw leaves	84.63 ± 0.70^a^	16.54 ± 0.77^a^	25.21 ± 2.63^a^	25.68 ± 2.36^b^	24.25 ± 2.33^a^
	boiled 30 min	95.67 ± 0.24^a^	14.27 ± 1.64^a^	25.94 ± 4.95^a^	46.69 ± 2.0^a^	0^b^
	boiled 60 min	92.57 ± 1.45^b^	18.20 ± 3.00^a^	26.75 ± 3.92^a^	47.72 ± 7.66^a^	0^b^
	*p*‐value	.0001	.460	.906	.002	.0001
	*t* test	**	ns	ns	**	**
Black nightshade	Raw leaves	87.13 ± 1.39^c^	10.57 ± 1.12^a^	39.83 ± 204^a^	26.94 ± 5.04^c^	16.39 ± 0.08a
	boiled 30 min	95.81 ± 056^a^	7.32 ± 0.87^a^	39.39 ± 2.12^a^	57.47 ± 020^a^	5.37 ± 0.18^b^
	boiled 60 min	93.44 ± 0.59^b^	8.80 ± 2.18^a^	32.28 ± 1.59^b^	50.54 ± 022^b^	2.30 ± 0.12^c^
	*p*‐value	.0001	.233	.006	.0001	.0001
	*t* test	**	ns	**	**	**
Jute mallow	Raw leaves	83.73 ± 1.64^c^	12.39 ± 1.44^a^	29.18 ± 0.47^a^	27.88 ± 4.36^ab^	27.71 ± 1.75^a^
	boiled 30 min	96.90 ± 063^a^	9.37 ± 2.66^a^	20.09 ± 022^c^	32.04 ± 3.36^a^	0^b^
	boiled 60 min	94.18 ± 0.32^b^	11.43 ± 0.70^a^	26.32 ± 0.69^b^	19.88 ± 3.36^b^	0^b^
	*p*‐value	.0001	.190	.0001	.102	.0001
	*t* test	**	ns	**	ns	**

Values in columns followed by the same letter are not significantly different.

ns, *, ** nonsignificant or significant at *p* < .05 or *p* < .01.

There was no change in protein content in amaranth, black nightshade and jute mallow leaves and black nightshade leaves boiled for 30 min. The protein content of jute mallow leaves had decreased by boiling for 30 min. There was no difference between protein content of raw and amaranth leaves boiled for 60 min. For black nightshade and jute mallow leaves protein content in raw leaves was higher than for those boiled for 60 min. Carbohydrate content increased through the boiling process for amaranth and black nightshade leaves after 30 and 60 min. There was no difference between carbohydrate content of raw and boiled leaves of jute mallow. There was a loss of β‐carotene content due to boiling. For amaranth and jute mallow leaves, no β‐carotene was detected in boiled leaves. For black nightshade leaves loss of β‐carotene was lower for 30 min of boiling than for 60 min.

Drying led to reduced leaf moisture content (Table 4). The loss was similar for all leaves dried in the shade and at 50 or 60°C. There were few variations in ash content in dried compared to raw leaves. The crude lipid content decreased with the drying process. There were no differences in crude lipid content in all crops regardless of drying method. There was no difference in protein content between raw and dried leaves of amaranth and black nightshade. For jute mallow there were decreases in protein content due to drying in the shade and drying at 50 or 60°C between raw and dried leaves. For all crops, carbohydrate content decreased during the drying processes. Drying reduced β‐carotene content of the leaves of all crops.

**Table 4:** Effect of drying on nutritional content of the vegetable (dry matter basis)

Crop ×	Condition	Moisture (%)	Ash (%)	Lipid (%)	Protein (%)	Carbohydrates (%)	β‐ carotene (mg/100 g)
Amaranth	Raw leaves	84.63 ± 0.70^a^	16.54 ± 0.77^a^	7.56 ± 1.37^a^	25.21 ± 2.63^a^	25.68 ± 2.36^a^	24.25 ± 2.33^b^
	Shade drying	11.82 ± 3.58^b^	16.43 ± 1.61^a^	3.96 ± 0.41^b^	23.24 ± 4.44^a^	13.97 ± 0.73^b^	0.63 ± 0.12^b^
	Cabin drying at 60°C	7.29 ± 2.14^b^	15.14 ± 1.17^a^	3.57 ± 0.39^b^	23.62 ± 0.82^a^	12.94 ± 2.09^b^	0.11 ± 0.00^b^
	Cabin drying at 50°C	7.38 ± 3.84^b^	16.31 ± 1.94^a^	3.64 ± 0.63^b^	24.10 ± 6.33^a^	10.20 ± 3.52^b^	0.19 ± 0.06^b^
	*p*‐value	.0001	.734	.004	.939	.000	.0001
	*t* test	**	ns	**	ns	**	**
Black nightshade	Raw leaves	87.71 ± 1.61^a^	10.57 ± 0.83^a^	7.11 ± 1.22^a^	39.74 ± 1.92^a^	26.94 ± 5.04^a^	16.39 ± 0.08^a^
	Shade drying	11.82 ± 3.5^b^	12.34 ± 1.75^a^	4.03 ± 0.92^b^	38.24 ± 1.63^a^	17.09 ± 1.86^b^	1.76 ± 0.00^b^
	Cabin drying at 60°C	6.47 ± 1.24^c^	11.51 ± 0.53^a^	4.05 ± 0.36^b^	36.34 ± 1.39^a^	13.98 ± 0.88^b^	1.09 ± 0.00^c^
	Cabin drying at 50°C	10.18 ± 3.98^bc^	11.67 ± 0.69^a^	4.14 ± 0.53^b^	36.07 ± 2.24^a^	16.60 ± 1.58^b^	1.11 ± 0.01^c^
	*p*‐value	.0001	.352	.001	.136	.006	.0001
	*t* test	**	ns	**	ns	**	**
Jute mallow	Raw leaves	83.73 ± 1.64^a^	12.39 ± 1.44^a^	6.64 ± 1.46^a^	29.18 ± 0.47^a^	27.88 ± 3.76^a^	27.71 ± 1.75^a^
	Shade drying	9.32 ± 1.42^b^	11.20 ± 1.04^a^	4.11 ± 0.94^b^	25.69 ± 0.76^b^	19.14 ± 5.86^a^	0.69 ± 0.01^b^
	Cabin drying at 60°C	7.09 ± 0.74^b^	7.96 ± 0.63^b^	5.32 ± 1.36^ab^	23.46 ± 1.26^b^	22.35 ± 3.25^ab^	0^b^
	Cabin drying at 50°C	7.48 ± 1.36^b^	11.96 ± 0.23^a^	4.59 ± 0.37^b^	24.39 ± 2.4^b^	23.58 ± 2.10^ab^	0^b^
	*p*‐value	.0001	.028	.049	.001	.136	.0001
	*t* test	**	*	*	**	ns	**

Values in columns followed by the same letter are not significantly different.

ns, *, ** nonsignificant or significant at *p* < .05 or *p* < .01.

## 4 DISCUSSION

Some nutritional content reported here are close to that reported by Odhav, Beekrum, Akula, and Baijnath (2007). Schönfeldt and Pretorius (2011) reported a lower moisture content for jute mallow leaves compared to values in the present study. These differences might be due to leaf maturity stage, environmental factors and harvesting method (Jain & Sutarno, 1996). Higher moisture content constitutes an alteration of leaves, which can lead to postharvest losses.

Idirs et al. (2009) reported different ash content for jute mallow leaves than found here. Differences in ash content may be due to leaf age at harvest, or the mineral fertilizer used in culture (Sossa‐Vihotogbe, 2013). Variation in ash content in vegetables can be due to cultural practices (Agbo, Kouamé, Mahyao, N'Zi, & Fondio, 2009; Nordeide, Hatloy, Folling, Lied, & Oshaug, 1996), soil mineral composition and the proportion of individual mineral absorption by each plant (Anjorin, Ikokoh, & Okolona, 2010; Asaolu & Asaolu, 2010). In this study, differences might be explained by diversity of sample collection site, or by the cultural practice of each farmer.

The crude lipid content for amaranth, black nightshade, and jute mallow leaves were higher than reported by Grubben et al. (2014). Idirs et al. (2009) found a higher crude lipid content in jute mallow leaves. The crude lipid content could make these crops contribute to meeting the daily lipid requirement of consumers. However, a higher crude lipid content would make the product highly susceptible to rancidity (Odibo, Ezeaku, & Ogbo, 2008).

The crops investigated are an important source of protein. For amaranth protein content was lower than reported by Ocho‐Anin, Lêniféré, Christophe, Edith, and Kouakou (2012) and Grubben et al. (2014). For black nightshade leaves, the average found here was lower than that reported by Ocho‐Anin et al. (2012). For jute mallow leaves, Idirs et al. (2009) and Grubben et al. (2014) reported lower protein values. Ocho‐Anin et al. (2012) reported higher protein levels of jute mallow leaves. The differences in protein content could be due to fertilizer used and soil composition (Agbo et al., 2009).

Carbohydrate content of leaves were lower than reported by Grubben et al. (2014) for amaranth, black nightshade and jute mallow leaves, or by Tchiegang and Kitikil (2004) for black nightshade leaves. Differences may be attributed to analysis method, plant age, farming and environmental conditions, plant species, or cultivar (Jain & Sutarno, 1996).

The crops are sources of β‐carotene. Various values of β‐carotene have been reported for amaranth leaves (Mosha, Gaga, Pace, Laswai, & Mtebe, 1997; Negi & Roy, 2001), black nightshade (Mibei & Ojijo, 2011) and jute mallow (Choudhary et al., 2013). The differences may be attributed to analysis methods (Jain & Sutarno, 1996). In previous works (Mibei & Ojijo, 2011; Mosha et al., 1997; Negi & Roy, 2001) β‐carotene was determined by titration while in the present study β‐carotene was determined by High Performance Liquid Chromatography.

Increase in moisture content in blanched leaves can be attributed to disruption of cell walls and membranes allowing water to fill spaces. Reduction in ash content can be explained by minerals leaching during blanching and cooling processes. Blanching led to increased protein content for amaranth and black nightshade leaves. Protein content of jute mallow decreased. This difference could be explained by the nature and form of protein of each specific leaf. Increase in carbohydrate content could be attributed to hydrolization of complex glucidic chains (Nafir‐Zenati, Gallon, & Faver, 1993) freeing sugar molecules. Blanching leaves induced loss of β‐carotene. The loss could be due to blanching temperature. The loss of β‐carotene in leaves were higher than those reported by Negi and Roy (2000) in amaranth leaves. Differences may be due to blanching time and use of potassium metabisulfite applied by others. Metabisulfite is used to retain leaf color and carotenoids compounds including β‐carotene. It is understandable that leaves that have been cooked for 30–60 min loose more compounds that those which have been blanched for 1 min.

Boiling leads to increased leaf moisture content because of water absorption during the cooking process. The lower ash content in boiled leaves compared to raw leaves could be explained by a transfer of minerals from leaves to the boiling water. Loss is less pronounced in leaves cooked for 30 than for 60 min. There was an increase in carbohydrates content of boiled leaves. These results may be explained by the hydrolyzation of glucidic polymers to free sugar molecules during boiling. (Nafir‐Zenati et al., 1993).

Boiling affected leaf β‐carotene content. Loss was more pronounced for amaranth and jute mallow than for black nightshade leaves. The loss can be attributed to temperature effect on β‐carotene. Chandler and Schwartz (1988) reported that loss of carotenoids during cooking is due to oxidation reactions and isomerization. They reported that during cooking treatment time is associated with transformation of the trans‐configurations of carotenoids to cis‐configuration, which are less active.

The reduction in moisture content of leaves during drying was due to evaporation by convection. Moisture content of leaves dried in a drying cabinet at 50 or 60°C was different than that reported by James and Kuipers (2003) for dried vegetable leaves. This is likely due to an insufficient drying (short time) or an inadequate drying method.

The drying process induced loss in β‐carotene content that can be attributed to drying temperature (Negi & Roy, 2000). They reported that drying at moderate temperature (30 ± 2°C) allowed for better retention of β‐carotene. This loss could be reduced by bleaching and sulfur treatment prior to drying (James & Kuipers, 2003).

Amaranth, black nightshade and jute mallow leaves have very high nutrient, mineral and β‐carotene potential. Postharvest processes affect nutritional content of these vegetables, particularly β‐carotene loss, which is pronounced following blanching and boiling of amaranth and jute mallow leaves. Black nightshade leave exhibited the highest β‐carotene retention during all processes. Protein and ash content were relatively stable regardless of the process. Blanching appeared to preserve more leaf nutrients. Time and temperature of treatments should be taken into account during postharvest processes to preserve nutrients. To minimize qualitative and nutritive losses, vegetables should be cooked for a shorter time, cooking in less water, and consuming the water used for cooking as a part of the diet.
